# Biosynthesis of Oligomeric Anthocyanins from Grape Skin Extracts

**DOI:** 10.3390/molecules22030497

**Published:** 2017-03-21

**Authors:** Jin-Woo Hwang, Sithranga Boopathy Natarajan, Yon-Suk Kim, Eun-Kyung Kim, Jae Woong Lee, Sang-Ho Moon, Byong-Tae Jeon, Pyo-Jam Park

**Affiliations:** 1 Korean Nokyong Research Center, Konkuk University, 380-701 Chungju, Korea; croucard@kku.ac.kr (J.-W.H.); kimyonsuk@kku.ac.kr (Y.-S.K.); eunkyungkim@kku.ac.kr (E.-K.K.); moon0204@kku.ac.kr (S.-H.M.); hannokwon@kku.ac.kr (B.-T.J.); 2 Department of Biotechnology, Konkuk University, 380-701 Chungju, Korea; nsboopathy@gmail.com (S.B.N.); ehdzld112@naver.com (J.W.L.); 3 Division of Food Bioscience, Konkuk University, 380-701 Chungju, Korea

**Keywords:** oligomeric anthocyanin, fermentation, crude enzyme, *Aspergillus niger*, glucosidase

## Abstract

We synthesized oligomeric anthocyanins from grape skin-derived monomeric anthocyanins such as anthocyanidin and proanthocyanidin by a fermentation technique using *Aspergillus niger*, crude enzymes and glucosidase. The biosyntheses of the oligomeric anthocyanins carried out by the conventional method using *Aspergillus niger* and crude enzymes were confirmed by ESI-MS. The molecular weight of the synthesized anthocyanin oligomers was determined using MALDI-MS. The yield of anthocyanin oligomers using crude enzymes was higher than that of the synthesis using *Aspergillus* fermentation. Several studies have been demonstrated that oligomeric anthocyanins have higher antioxidant activity than monomeric anthocyanins. Fermentation-based synthesis of oligomeric anthocyanins is an alternative way of producing useful anthocyanins that could support the food industry.

## 1. Introduction

Anthocyanins are naturally occurring water-soluble plant pigments belonging to the group of phytochemicals known as flavonoids [1]. Anthocyanins are present in many plants which display colorful flowers, and different kinds of fruits and vegetables [2,3,4]. The quality and nutritional value of fruits and their products is commonly associated with the color that is derived from anthocyanins [5,6]. Anthocyanins are very useful for the food industry, due to their good water solubility and safety. They have been recognized internationally for their applications, including the replacement of synthetic colorants [7,8]. Anthocyanins have antioxidant activity which contributes to many biological activities such as anticancer, cardiovascular protection, ocular protection and protection against some other chronic diseases [9,10,11,12]. Several studies have been demonstrated that the oligomeric derivatives of anthocyanin have higher activity than the monomeric versions. For example, the anthocyanin oligomers derived from bilberry fruit such as small anthocyanidin glycoside polymers, particularly in the form of dimers, trimers, tetramers and pentamers have higher antioxidant activity than the monomers. These compounds are highly hydro- and liposoluble in nature and are not known to accumulate in the human body [13].

The biosynthesis of oligomeric anthocyanins is the best alternative to overcome the problem of deficiency. At present, studies on the synthesis of anthocyanin oligomers are scarce, and only one related paper is available [13]. *Aspergillus* species such as *Aspergillus niger*, *A. sojae* and *A. oryzae* have long been used for the production of traditional fermented foods such as doenjang, cheonggukjang, soy sauce and sake in Asian countries [14,15]. Fungi are rich sources of citric acid [16], C_8_ volatiles [14] and many enzymes such as xylanase, cellulose [17], amyloglucosidase and exopolygalacturonase [18]. However, in the industrial applications of these fungi overcoming their contamination is a big challenge. The present study focuses on the synthesis of oligomeric anthocyanins by fermentation of monomeric anthocyanins with *Aspergillus niger*, as well as with crude enzymes derived from the fungus.

## 2. Results and Discussion

**Figure 1:**
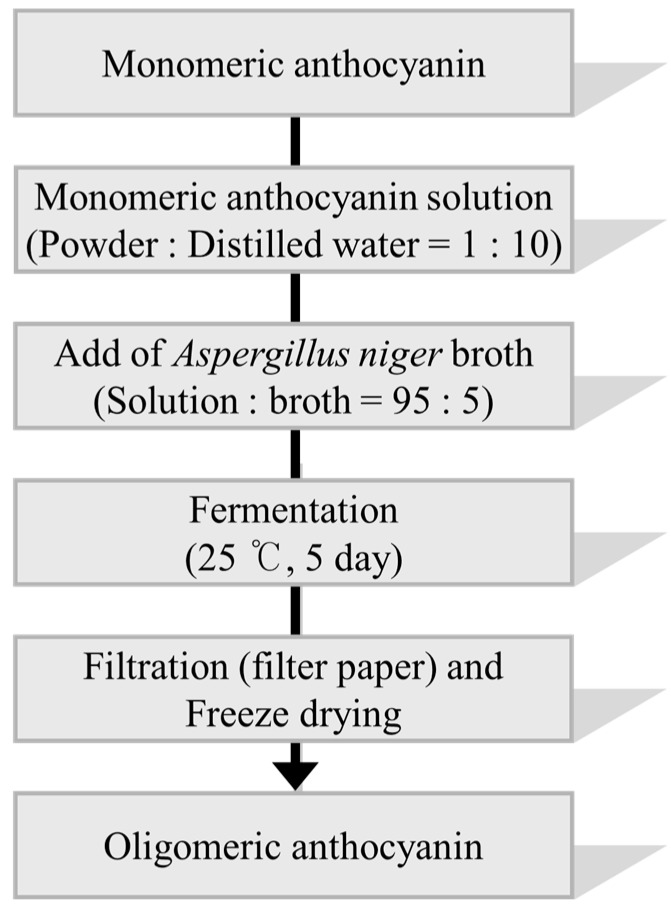
The flow chart of oligomeric anthocyanin synthesis using *Aspergillus niger*.

**Figure 2:**
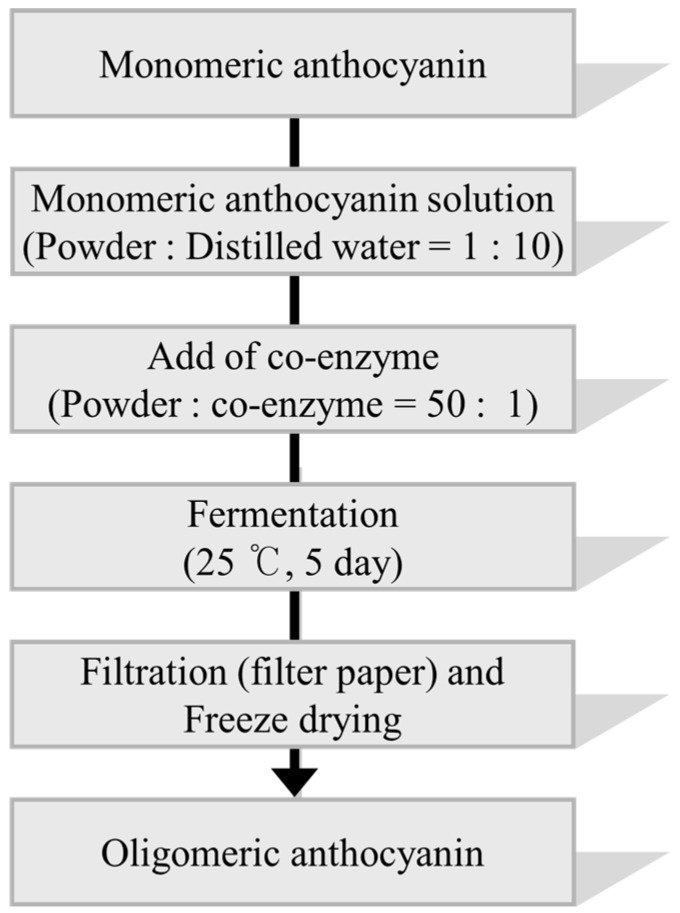
The flow chart for the synthesis of oligomeric anthocyanins using crude enzyme from *Aspergillus niger*.

**Figure 3:**
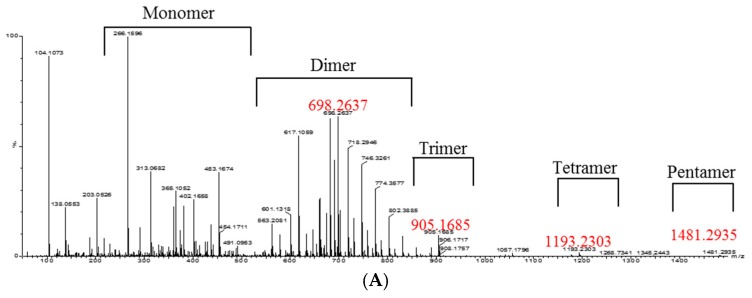

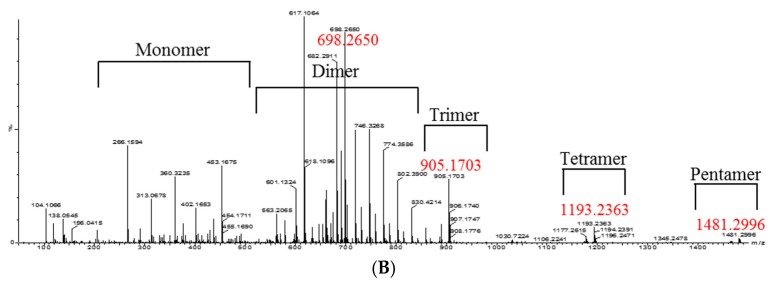
The ESI-MS observation of synthesis of oligomeric anthocyanins from monomeric anthocyanin using *Aspergillus niger*. (**A**) Before biosynthesis; (**B**) after biosynthesis.

**Figure 4:**
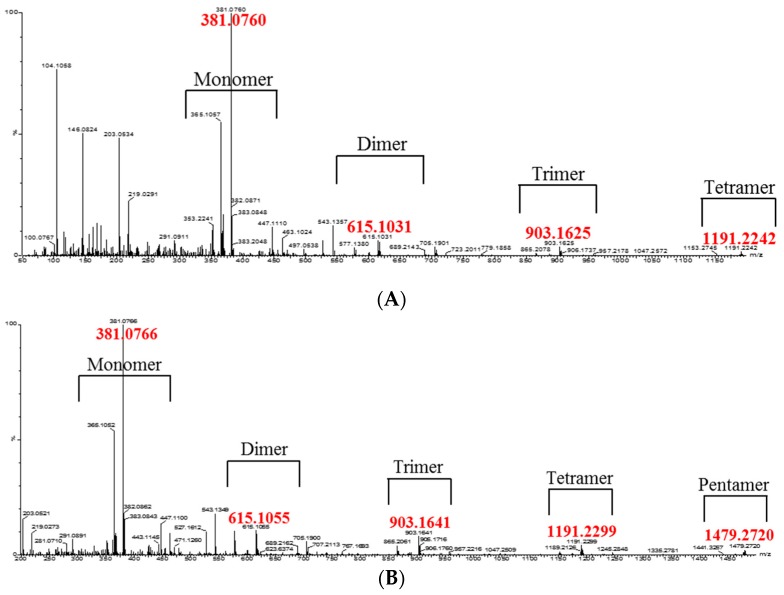
The synthesis of oligomeric anthocyanins from another monomeric anthocyanin (proanthocyanidin) using crude enzyme derived from *Aspergillus niger*. (**A**) Before biosynthesis; (**B**) after biosynthesis.

The oligomeric anthocyanins were successfully synthesized by fermentation using *Aspergillus niger* (Figure 1) as well as crude enzyme (Figure 2) as confirmed by ESI-MS (Figure 3 and Figure 4). The oligomeric anthocyanins showed higher peak values and higher molecular weight than the monomeric anthocyanins. The higher peak value might be attributed to the presence of higher amount of oligomeric anthocyanins under similar experimental conditions [19]. It was confirmed that the yield of oligomeric anthocyanins derived from the fermentation by crude enzyme was better than that derived from the fermentation with *Aspergillus niger* (Table 1).

We have previously reported the synthesis and characterization of anthocyanin oligomers produced by *A. niger* fermentation using anthocyanin monomers as substrate [13]. The molecular weight of the anthocyanin oligomers was determined using Matrix Assisted Laser Desorption/Ionization Mass Spectrometry (MALDI-MS) [20]. In this study, the biosynthesis of oligomeric anthocyanins was detected using the relative absorbance values of compounds estimated by ESI-MS. ESI-MS is an important technique to detect femtomole quantities of sample, including non-volatile and thermally labile biomolecules that are difficult to analysis by other conventional techniques [21]. Liu et al. [22] detected the monomers, dimers, tetramers and hexamers of purified oligomeric proanthocyanins using ESI-MS. Therefore, the present study also used ESI-MS to analyze the various structures of the oligomeric proanthocyanins.

The monomeric anthocyanins such as anthocyanidin and proanthocyanidin give peaks at 288 *m*/*z* (Figure 3A) and 381 *m*/*z* (Figure 4A), respectively. The oligomeric anthocyanins synthesized from anthocyanidin monomers showed the peak values of *m*/*z* 905, 1193 (Figure 3B). Similarly, the oligomeric anthocyanins synthesized from the other monomer (proanthocyanidin) showed the following highest peak values: *m*/*z* 903, 1191, 1479 (Figure 4B). Therefore, the differentiation of peak values between before and after fermentation confirmed the synthesis of oligomeric anthocyanins using crude enzyme as well as fermentation with *Aspergillus niger*. The amount of oligomeric anthocyanin synthesized from fermented crude enzyme was higher than that synthesized from fermentation with *Aspergillus niger*.

**Figure 5:**
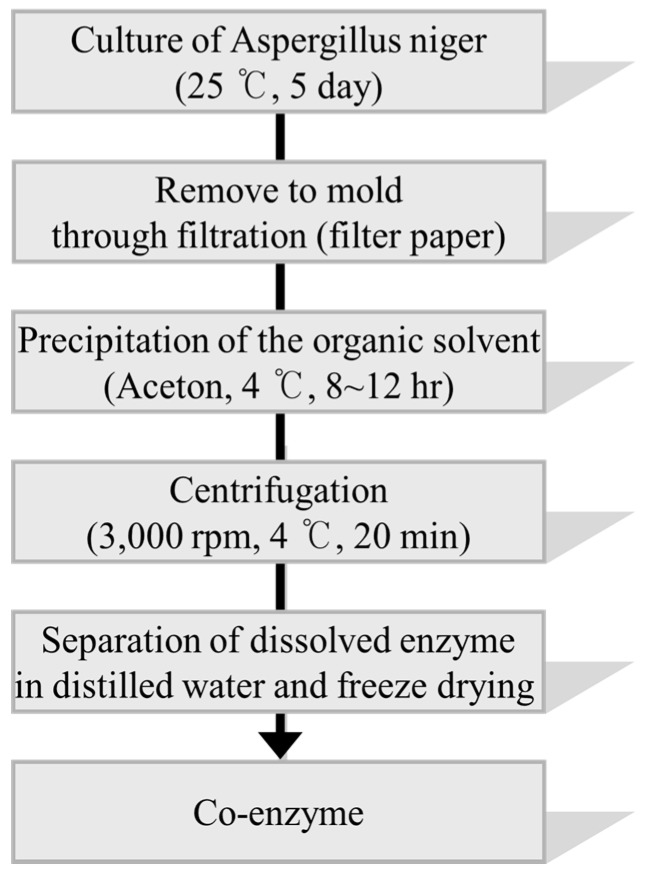
The flowchart of method to obtain the crude enzyme from *Aspergillus niger*.

**Figure 6:**
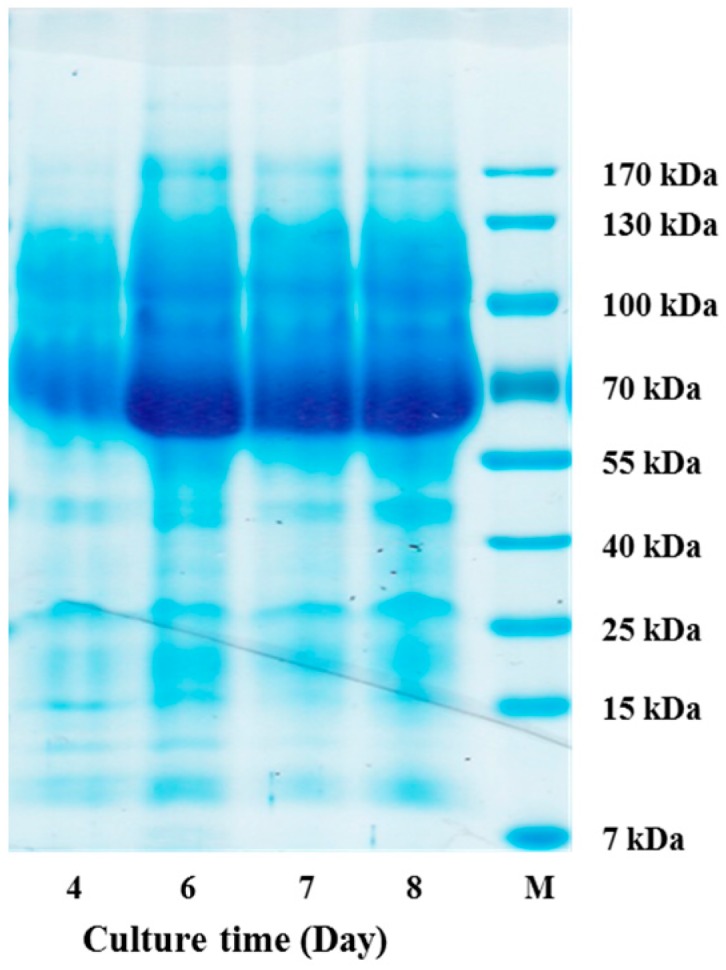
Confirmation of the crude enzyme using SDS-PAGE for each culture day.

**Figure 7:**
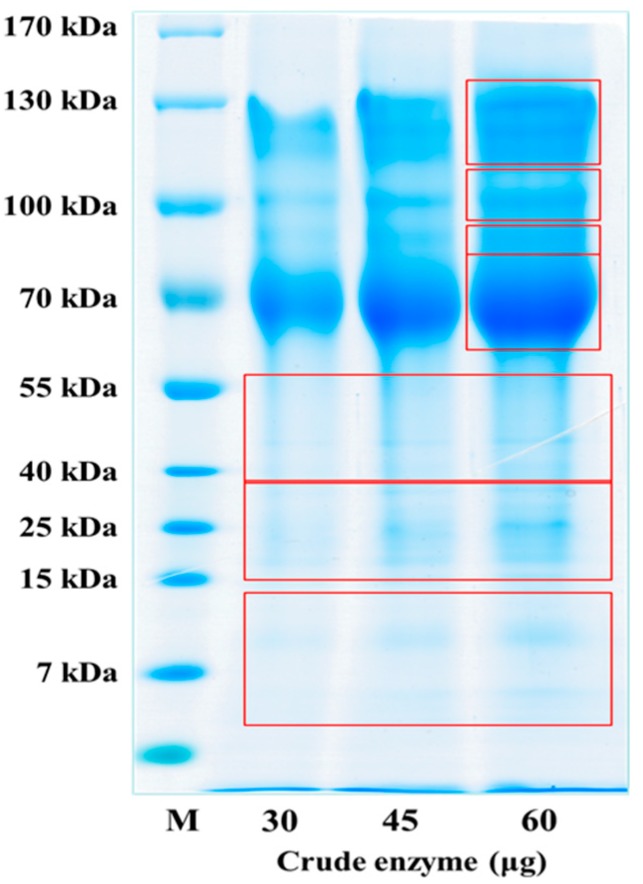
Separation of co-enzyme using SDS-PAGE.

**Figure 8:**
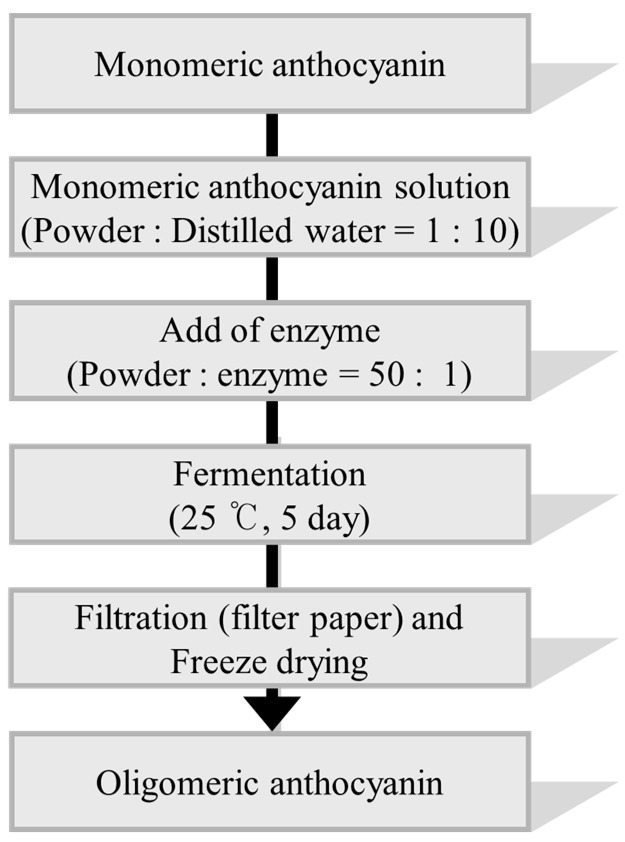
The flow chart for the synthesis of oligomeric anthocyanins using glucosidase from *Aspergillus niger*.

**Figure 9:**
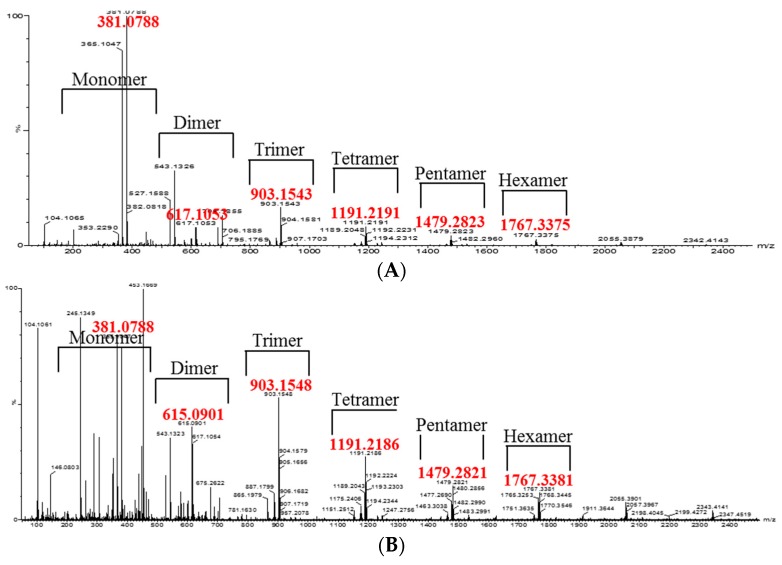
The synthesis of oligomeric anthocyanin from monomeric anthocyanin using glucosidase from *Aspergillus niger*. (**A**) Before biosynthesis; (**B**) after biosynthesis.

**Figure 10:**
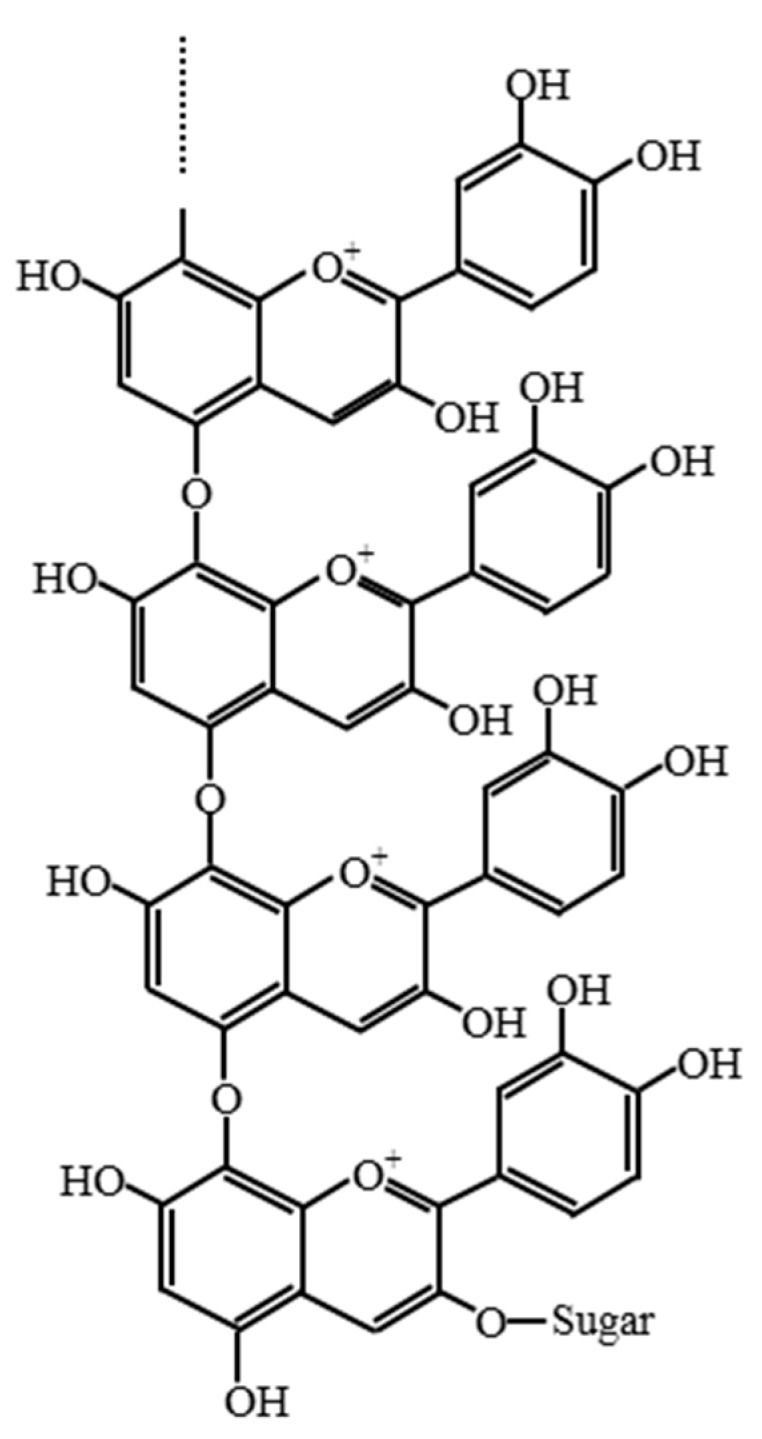
The structure of oligomeric anthocyanin.

The enzyme for the synthesis of oligomeric anthocyanin (Figure 5 and Figure 8) was electrophoresed by SDS-PAGE (Figure 6 and Figure 7) and the tryptic peptides obtained from each gel slice (Figure 7) were analyzed by LC-MS/MS run on a Q-STAR Pulsar ESI-hybrid Q-TOF instrument (Table 2). According our literature search, some of the carbohydrate hydrolases mentioned in Table 2 were found to produce condensation reactions [23]. After the synthesis (Figure 8), the content of anthocyanin was determined based on the presence of glucosidase in the product (Figure 9). The results demonstrate the similarity of pattern between Figure 3 and Figure 4. Figure 9 indicates a difference in molecular weight of *m*/*z* 288 for each oligomeric anthocyanin peak, which corresponds to the molecular weight of cyanidin. Consequently, the structure of anthocyanins was presumed to be as shown in Figure 10.

Comparing the yield of the oligomeric anthocyanins, it was seen that the highest yield was obtained using crude enzyme (Table 1), but crude enzymes are difficult to obtain. Therefore, it was thought that it might be more economical to use a commercially available glucosidase. In addition, as seen in Figure 4B and Figure 9B, the oligomeric anthocyanins synthesized with glucosidase showed higher oligomer content than oligomeric anthocyanin synthesized with crude enzyme.

**Figure 11:**
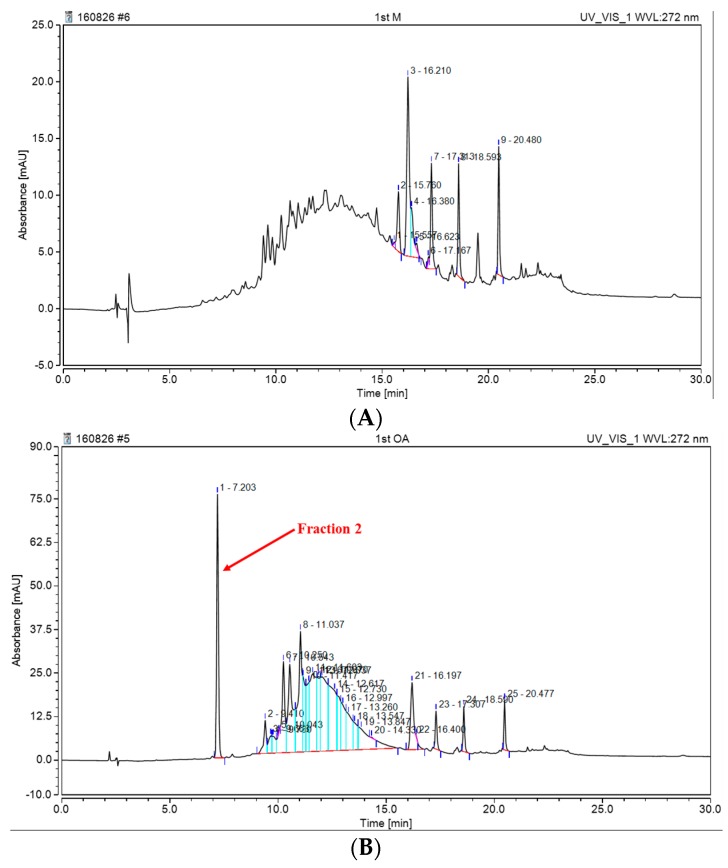
HPLC patterns of monomeric and oligomeric anthocyanins. (**A**) Before biosynthesis; (**B**) after biosynthesis using glucosidase.

**Figure 12:**
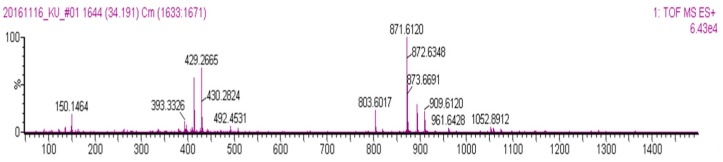
LC/MS analysis of two fractions of the oligomeric anthocyanin.

**Figure 13:**
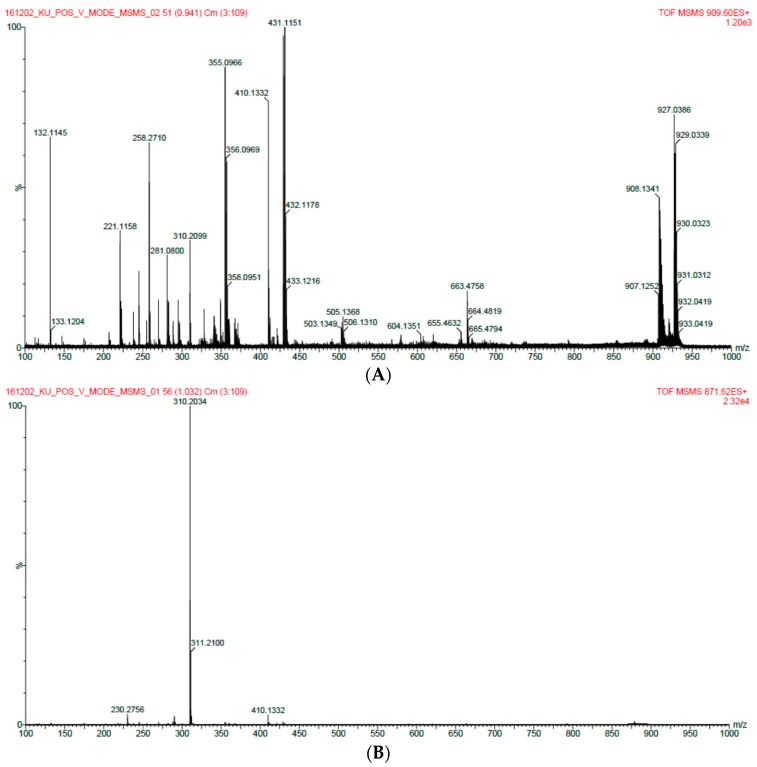
ESI-MS analysis of 871 *m*/*z* material. (**A**) *m*/*z* 871 peak of the LC/MS analysis results; (**B**) *m*/*z* 927 peak of the ESI-MS analysis results.

The comparison of monomeric and oligomeric anthocyanins using HPLC with UV detection confirmed that the fractions have different patterns (Figure 11). Different patterns were fractionated and analyzed by LC/MS. In the second fraction, a single substance showing a molecular weight of *m*/*z* 429, 871 was identified at 7 min (Figure 12). It was assumed that the compound with molecular weight *m*/*z* 871 was the dimer and that with *m*/*z* 429 was the monomer. This assumption was correlated to the results obtained by ESI-MS for the *m*/*z* 871 peak (Figure 13A). Based on the results of Figure 13, the *m*/*z* 429 compound consisted of anthocyanidin (*m*/*z* 310) and glucose. The *m*/*z* 871 peak is the dimeric form of the *m*/*z* 429 species. An NMR study is needed for better understanding of these molecular structures.

In summary, the biosynthesis of oligomeric anthocyanins using fermentation is an alternative approach to overcome the problem of their natural scarcity to avoid the overexploitation of natural resources.

## 3. Materials and Methods

### 3.1. Materials

5-Dimethyl-1-pyrroline-*N*-oxide (DMPO), FeSO_4_, and H_2_O_2_ were purchased from Sigma Chemical Co. (St. Louis, MO, USA). KH_2_PO_4_, KCl and NaCl were purchased from Junsei (Tokyo, Japan). Saccharose, dextrose, urea, MgSO_4_, MnSO_4_, and ZnSO_4_ were purchased from Deajung (Siheung, Korea). Peptone G was purchased from Acumedia (Lansing, MI, USA). The grape skin- derived anthocyanins were purchased from Kitolife Co. Ltd. (Pyeongtaek, Korea).

### 3.2. Culture Medium of Aspergillus niger

The *Aspergillus niger* culture was maintained at 25 °C in a shaking incubator. The culture media contained saccharose (60 g/L), peptone G (10 g/L), dextrose (5 g/L), urea (0.5 g/L), MgSO_4_ (0.25 g/L), KH_2_PO_4_ (0.04 g/L), KCl (0.075 g/L), NaCl (0.075 g/L), MnSO_4_ (0.01 g/L) and ZnSO_4_ (0.005 g/L).

### 3.3. Synthesis of Oligomeric Anthocyanin by Fermentation Using Aspergillus niger

Synthesis of oligomeric anthocyanins using *Aspergillus niger* was described by Lee et al. [13]. In this study, monomeric anthocyanins such as anthocyanindins and proanthocyanidins were used to synthesize oligomeric anthocyanins. The monomeric anthocyanin powders were fermented with *Aspergillus niger* at 25 °C in a shaking incubator for 5 days. The fermented cultures were centrifuged at 3000 rpm, 4 °C, for 20 min. The supernatants were filtered with Whatman No. 41 filter paper and the filtrate was freeze-dried by a freeze drier system (SFDSM06, Samwon, Busan, Korea) in order to obtain the synthesized oligomeric anthocyanins. The concentrations of fermented oligomeric anthocyanin produced by fermentation were estimated using Electrospray Ionization-Mass Spectrometry (ESI-MS) at the Korea Basic Science Institute (KBSI, Ochang, Korea). The molecular mass values of the compounds were analyzed by a Synapt G2 HDMS quadrupole time-of-flight (TOF) mass spectrometer equipped with an electrospray ion source (Waters, Milford, MA, UK) in positive ion mode at a spray voltage of 2.5 kV. MS spectra were obtained with the capillary heated to 150 °C. The instrument was calibrated using NaF solution. The sample was dissolved in 100% MeOH and introduced by direct infusion at a flow rate of 20 μL/min into the ion source operating in positive mode. All spectra were acquired at a range of 50 to 2500 *m*/*z*. Leucine enkephalin was used as the lock mass for the exact mass measurement correction.

### 3.4. Separation of Fermented Crude Enzyme from the Culture of Aspergillus niger

The fungal strain *Aspergillus niger* was cultured in 100 mL saccharose medium or potato dextrose agar (PDA) medium and incubated at 25 °C, for 7 days in an shaking incubator. The *Aspergillus niger* culture medium was centrifuged at 3000 rpm, 4 °C, for 20 min. The supernatant was precipitated with an equal volume of acetone at 4 °C, overnight (10–12 h) and this mixture was then centrifuged at 3000 rpm, 4 °C for 20 min. After removal of the supernatant, the pellet was dissolved using 5 mL of distilled water and further centrifuged at 13,000 rpm, 4 °C, for 5 min. The supernatant (crude enzyme) was freeze-dried in order to synthesize the oligomeric anthocyanin.

### 3.5. Synthesis of Oligomeric Anthocyanin Using Crude Enzyme

The anthocyanin powder was fermented with crude enzyme at 25 °C in a shaking incubator for 7 days. The fermented stuff was centrifuged at 4 °C, 3000 rpm for 20 min. The supernatant was filtered with Whatman No. 41 filter paper and the filtrate was freeze-dried in a SFDSM06 freeze drier system in order to obtain the synthesized oligomeric anthocyanins. The concentration of oligomeric anthocyanin was examined by ESI-MS at KBSI.

### 3.6. Analysis of Crude Enzyme from the Culture of Aspergillus niger

SDS-PAGE gel slicing was used for LC-MS/MS analysis of the secretory proteins from *Aspergillus niger*. The soluble proteins in urea lysis buffer containing 8 M urea and 4% CHAPS after the acetone precipitation of the secretory fraction from *Aspergillus niger* was subjected to 6–15% SDS-PAGE and stained with colloidal Coomassie solution. The lanes, which are cut as nine slices, were excised from the gel in three of the protein lanes for the mass spectrometry experiments by destaining and in-gel digestion followed by peptide extraction. The tryptic peptides obtained from each gel slice were analyzed by LC-MS/MS running on the Q-STAR Pulsar ESI-hybrid Q-TOF instrument.

### 3.7. Synthesis of Oligomeric Anthocyanin Using Various Enzymes

The anthocyanin powder was fermented with various enzymes at 25 °C in a shaking incubator for 7 days. The fermented product was centrifuged at 4 °C, 3000 rpm for 20 min. The supernatant was filtered with Whatman No. 41 filter paper and the filtrate was freeze-dried by a freeze dry system (SFDSM06) in order to obtain the synthesized oligomeric anthocyanins The concentration of oligomeric anthocyanin was examined by ESI-MS at KBSI.

### 3.8. Isolation of Oligomeric Anthocyanin and Analysis

The monomeric and oligomeric anthocyanins were further isolated using reversed-phase HPLC (RP-HPLC) on a C_18_ column (4.0 × 250 mm) with a linear gradient of MeOH (0–60%) at a flow rate of 1.0 mL/min. The eluted peaks were detected at 272 nm. The collected samples were pooled and concentrated using a rotary evaporator, then lyophilized for 3 days. The lyophilized sample was further analyzed by LC/MS followed by ESI-MS at KBSI.

### 3.9. Statistical Analysis

The statistical analyses was carried out by the paired *t*-test (*p* < 0.05) and comparisons made between monomeric anthocyanins and oligomeric anthocyanins. The data are presented as mean ± SD. All analyses were performed using the SPSS software (SPSS Institute, Chicago, IL, USA).

## 4. Conclusions

Our results indicate that the synthesis of oligomeric anthocyanins using glucosidase from *A. niger* is better than that possible with fermentation of *A. niger*. Synthesis of oligomeric anthocyanins was confirmed by ESI-MS and HPLC analysis. The present study successfully overcome the problem of fungal contamination during synthesis of oligomeric anthocyanins. Further studies are however required to assess the biological activities of the produced oligomeric anthocyanins.

## Figures and Tables

**Table 1:** The yield of oligomeric anthocyanins derived by fermentation as well as crude enzyme.

Fermentation	Monomeric Anthocyanin (g)	Oligomeric Anthocyanin (g)	Yield (%)
Aspergillus niger	10.1254	7.9835	78.27 ± 1.99 ^a^
	5.0354	4.0235	
	1.0024	0.7624	
Crude enzyme	10.0038	8.7685	87.93 ± 0.36 ^b^
	2.1539	1.9025	
	1.0657	0.9357	
Glucosidase	10.0387	8.5168	85.11 ± 0.45 ^b^
	5.1348	4.3578	
	1.0222	0.8753	

Different letters indicate significant different (*p* < 0.05) among samples as determined by the *t*-test. Values are the mean ± SD of triplicate determinations.

**Table 2:** Identification of proteins recovered from the secretory proteins from *Aspergillus niger*.

Gene I.D.	Protein Name	Probability	Molecular Weight
gi|224027	Glucoamylase G1	627	65,448
gi|134081727	Unnamed protein product [*Aspergillus niger*]	274	75,190
gi|765328	Acid phosphatase, orthophosphoric monoester phosphohydrolase, APase (EC 3.1.3.2) [*Aspergillus ficuum*, NRRL 3135, Peptide, 583 aa]	265	64,211
gi|257187	α-Glucosidase P2 subunit, ANP P2 subunit {EC 3.2.1.20} [*Aspergillus niger*, Peptide, 719 aa]	181	79,656
gi|2344	Preproglucoamylase G2 [*Aspergillus niger*]	531	56,695
gi|145242978	Hypothetical protein ANI_1_1546094 [*Aspergillus niger* CBS 513.88]	351	59,208
gi|145231236	Phospholipase C PLC-C [*Aspergillus niger* CBS 513.88]	410	49,652
gi|145235505	Serine carboxypeptidase [*Aspergillus niger* CBS 513.88]	297	62,560
gi|145252338	Phosphatidylglycerol specific phospholipase [*Aspergillus niger* CBS 513.88]	261	53,895
gi|4185610	Phytase [*Aspergillus niger*]	218	50,997
gi|145241119	3-Phytase B [*Aspergillus niger* CBS 513.88]	256	52,453
gi|145241490	1,3-β-Glucanosyltransferase gel3 [*Aspergillus niger* CBS 513.88]	161	56,721
gi|83655609	Acid phosphatase [*Aspergillus niger*]	142	52,725
gi|145242970	Hypothetical protein ANI_1_1540094 [*Aspergillus niger* CBS 513.88]	128	45,753
gi|145256696	Protein ecm33 [*Aspergillus niger* CBS 513.88]	125	41,026
gi|317026828	Serine-type carboxypeptidase F [*Aspergillus niger* CBS 513.88]	118	57,756
gi|145248273	Polyamine oxidase [*Aspergillus niger* CBS 513.88]	110	58,728
gi|145248205	Aspartic-type endopeptidase opsB [*Aspergillus niger* CBS 513.88]	104	50,958
gi|145234270	Glutaminase GtaA [*Aspergillus niger* CBS 513.88]	99	75,470
gi|350633205	Hypothetical protein ASPNIDRAFT_55058 [*Aspergillus niger* ATCC 1015]	87	22,487
gi|350631594	Hypothetical protein ASPNIDRAFT_53033 [*Aspergillus niger* ATCC 1015]	63	57,162
gi|145235707	FAD binding domain protein [*Aspergillus niger* CBS 513.88]	59	61,292
gi|145233743	α-Galactosidase B [*Aspergillus niger* CBS 513.88]	392	48,796
gi|317031802	Histidine acid phosphatase [*Aspergillus niger* CBS 513.88]	153	53,047
gi|317025164	Aspartic endopeptidase (AP1) [*Aspergillus niger* CBS 513.88]	483	46,701
gi|145242664	Sulphydryl oxidase [*Aspergillus niger* CBS 513.88]	264	43,471
gi|74626383	RecName: Probable α-galactosidase B; AltName: Melibiase B; Flags: Precursor	175	48,753
gi|134083538	Unnamed protein product [*Aspergillus niger*]	173	45,226
gi|400801	RecName: Pectin lyase A; Short=PLA; AltName: Full-Pectinlyase II; Short=PLII; Flags: Precursor	135	39,830
gi|145235303	Hypothetical protein ANI_1_496034 [*Aspergillus niger* CBS 513.88]	103	52,301
gi|134055991	Unnamed protein product [*Aspergillus niger*]	85	41,620
gi|134076313	Unnamed protein product [*Aspergillus niger*]	85	45,581
gi|145251519	Phosphoglycerate mutase family protein [*Aspergillus niger* CBS 513.88]	79	19,282
gi|350633205	Hypothetical protein ASPNIDRAFT_55058 [*Aspergillus niger* ATCC 1015]	73	22,487
gi|145232359	Endopolygalacturonase C [*Aspergillus niger* CBS 513.88]	241	37,796
gi|145235523	Glucan endo-1,3-β-glucosidase eglC [*Aspergillus niger* CBS 513.88]	129	46,778
gi|145230419	Glycosidase crf1 [*Aspergillus niger* CBS 513.88]	107	39,862
gi|129935	RecName: Full=Endopolygalacturonase II; Short=EPG-II; AltName: Full=Pectinase 2; AltName: Full=PolygalacturonaseII; Short=PG-II; AltName: Full=Polygalacturonase X2; Flags: Precursor	89	37,489
gi|133176	RecName: Full=Ribonuclease M; Short=RNase M	89	26,590
gi|134055750	Unnamed protein product [*Aspergillus niger*]	84	27,072
gi|145229151	Endo-1,3(4)-β-glucanase [*Aspergillus niger* CBS 513.88]	83	46,311
gi|134075575	Hypothetical protein An07g00170 [*Aspergillus niger*]	69	90,993
gi|134083538	Unnamed protein product [*Aspergillus niger*]	67	45,226
gi|145252266	GPI anchored cell wall protein [*Aspergillus niger* CBS 513.88]	64	19,022
gi|83638302	Xylanase [*Aspergillus phoenicis*]	117	10,944
gi|350633205	Hypothetical protein ASPNIDRAFT_55058 [*Aspergillus niger* ATCC 1015]	92	22,487
